# Recombinant human N-acetylgalactosamine-6-sulfate sulfatase (GALNS) produced in the methylotrophic yeast *Pichia pastoris*

**DOI:** 10.1038/srep29329

**Published:** 2016-07-05

**Authors:** Alexander Rodríguez-López, Carlos J. Alméciga-Díaz, Jhonnathan Sánchez, Jefferson Moreno, Laura Beltran, Dennis Díaz, Andrea Pardo, Aura María Ramírez, Angela J. Espejo-Mojica, Luisa Pimentel, Luis A. Barrera

**Affiliations:** 1 Institute for the Study of Inborn Errors of Metabolism, School of Sciences, Pontificia Universidad Javeriana, Bogotá, Colombia; 2 Chemical Department, School of Science, Pontificia Universidad Javeriana, Bogotá, Colombia

## Abstract

Mucopolysaccharidosis IV A (MPS IV A, Morquio A disease) is a lysosomal storage disease (LSD) produced by mutations on N-acetylgalactosamine-6-sulfate sulfatase (GALNS). Recently an enzyme replacement therapy (ERT) for this disease was approved using a recombinant enzyme produced in CHO cells. Previously, we reported the production of an active GALNS enzyme in *Escherichia coli* that showed similar stability properties to that of a recombinant mammalian enzyme though it was not taken-up by culture cells. In this study, we showed the production of the human recombinant GALNS in the methylotrophic yeast *Pichia pastoris* GS115 (prGALNS). We observed that removal of native signal peptide and co-expression with human formylglycine-generating enzyme (SUMF1) allowed an improvement of 4.5-fold in the specific GALNS activity. prGALNS enzyme showed a high stability at 4 °C, while the activity was markedly reduced at 37 and 45 °C. It was noteworthy that prGALNS was taken-up by HEK293 cells and human skin fibroblasts in a dose-dependent manner through a process potentially mediated by an endocytic pathway, without any additional protein or host modification. The results show the potential of *P. pastoris* in the production of a human recombinant GALNS for the development of an ERT for Morquio A.

Mucopolysaccharidosis IV A (MPS IV A, Morquio A disease, OMIM 253000) is a lysosomal storage disease (LSD) produced by mutations on N-acetylgalactosamine-6-sulfate sulfatase (GALNS, EC 3.1.6.4), leading to the lysosomal accumulation of keratan- and chondroitin-sulfate^1^,^2^. MPS IV A patients are characterized by short stature, hypoplasia of the odontoid process, pectus carinatum, kyphoscoliosis, genu valgum, laxity of joints, and corneal clouding, without central nervous system impairment^1^. MPS IV A patients are treated through non-steroidal anti-inflammatory drugs, antibiotics, oxygen supplementation, orthopedic surgical procedures, and hematopoietic stem cell transplantation^3^. Gene therapy has showed promising results in pre-clinical trials^4^,^5^,^6^, but further studies are necessary before it can be translated to patients.

Recombinant GALNS has been produced in CHO cells, showing an optimal pH ~5.0; the presence of 57, 39 and 19 kDa polypeptides; and the cellular uptake by cultured fibroblast and chondrocytes^7^,^8^. Pre-clinical trials showed that infusion of recombinant GALNS in MPS IV A animal models allowed a significant reduction of GAG storage in several tissues^7^,^9^. Recently an enzyme replacement therapy (ERT) for Morquio A disease was approved in Europe and USA using a recombinant enzyme produced in CHO cells. Patients receiving a 2.0 mg/kg/week dose showed a modest improvement in a 6-min walk test (6MWT) and a reduction of urinary KS, after 24 week treatment^10^. Furthermore, positive changes were observed in maximal voluntary ventilation, MPS-Health Assessment Questionnaire (MPS-HAQ), and height/growth rate^11^. Although ERT of elosulfase alfa is a therapeutic option for MPS IVA patients, current limitations include i) a limited effect on skeletal, corneal, and heart valvular issues^12^,^13^, ii) a short half-life of the enzyme and rapid clearance from the circulation, iii) immunological issues^14^, and iv) a high cost. An improved ERT with a long circulating enzyme and a bone-targeting enzyme have been proposed^9^, and a recombinant GALNS produced in other sources, may potentially help to resolve the above issues.

A growing number of studies have shown the possibility to produce active and therapeutic forms of lysosomal proteins in microorganisms^15^. The human lysosomal enzymes deficient in Gaucher, Fabry, Hunter, Pompe, α Mannosidosis, GM2 gangliosidosis, and acid lipase deficiency diseases have been produced in *Escherichia coli, Saccharomyces cerevisiae, Pichia pastoris* (currently reclassified as *Komagataella pastoris*)*, Yarrowia lipolytica,* and *Ogataea minuta*^16^,^17^,^18^,^19^,^20^,^21^,^22^,^23^,^24^. Among these hosts, yeasts represent an important platform for the production of recombinant proteins, since they can grow into economic culture media, are easily manipulated, secrete the recombinant protein to the medium, and produce heterologous proteins with similar post-translational modifications to those observed in human proteins^25^. Although yeast N-glycosylations have a different pattern (i.e. size and/or composition) to that observed in human proteins, these N-glycosylations can be glyco-engineered to produce tailored or homogeneous N-glycosylations^26^. Nevertheless, recombinant lysosomal human β-hexosaminidases^23^,^27^, α-glucosidase^18^, and lysosomal acid lipase^24^,^28^, produced in *P. pastoris*, have shown a dose-dependent cell uptake without any additional processing of the N-glycosylations.

Previously, we reported the production of a Recombinant GALNS enzyme in *E. coli* BL21(DE3) (erGALNS)^29^,^30^,^31^. Under batch culture conditions at 3L scale, the largest amount of erGALNS was obtained as inclusion bodies (~71%)^29^, while under semi-continuous culture conditions it was favoured the secretion of the recombinant enzyme^30^,^31^. Purified erGALNS showed a specific activity of 0.29 nmol h^−1^ mg^−1^ and a production yield of 0.78 mg per culture liter. In addition, erGALNS showed an optimal pH of 5.5 and similar temperature and serum stability profiles than those observed for GALNS produced in CHO cells^30^. However, this recombinant enzyme was not taken-up neither by HEK293 cells nor by Morquio A skin fibroblasts. Taken together, these results suggest that N-glycosylations in GALNS are not necessary for producing an active and stable enzyme, but they are for the protein cellular uptake^30^.

In this study, we evaluated the production of recombinant GALNS in the methylotrophic yeast *Pichia pastoris* GS115 (prGALNS), to produce a recombinant glycosylated version of GALNS using a microorganism host. Production was evaluated at 10, 100 and 1,650 mL scale, with or without the co-expression of the formylglycine-generating enzyme gene (*SUMF1*). prGALNS was purified from extracellular crude extract and the purified enzyme was characterized by pH and temperature stability, and *in-vitro* cellular up-take. The results show the potential of the recombinant GALNS produced in *P. pastoris* for the development of an ERT for Morquio A.

## Results

### Production of prGALNS at shake and bioreactor scales

Vectors pPIC9-GALNS and pPIC9-nspGALNS (GALNS cDNA without native signal peptide coding sequence) were digested with enzyme *Pme*I, which linearized the vectors by restriction at the 5′-AOX1 promoter fragment, to promote the gene insertion at the AOX1 loci. Five and three *P. pastoris* GS115 clones were obtained after transformation with pPIC9-GALNS or pPIC9-nspGALNS vectors, respectively. The presence of GALNS and nspGALNS cDNAs was confirmed in all the clones by PCR amplification. Since vectors were linearized with *Pme*I enzyme, all the evaluated clones had a Mut^+^ phenotype, as confirmed by PCR amplification of AOX1 locus.

All clones were screened at 10 mL scale and the production of recombinant GALNS was monitored during 96 h after induction with 0.5% methanol. At this scale, four out of five pPIC9-GALNS clones showed extracellular GALNS activity after the 24 h of induction, reaching a maximum enzyme activity of 0.024 (96 h), 0.048 (96 h), 0.034 (96 h) and 0.071 (72 h) U mg^−1^, for clones 2 to 5, respectively. On the other hand, all three pPIC9-nspGALNS clones showed GALNS activity at 10 mL scale with maximum values of 0.39 (24 h), 0.10 (24 h), and 0.057 (72 h) U mg^−1^, for clones 1 to 3, respectively. GALNS activity was not observed in *P. pastoris* GS115 transformed with empty pPIC9 vector.

**Figure 1:**
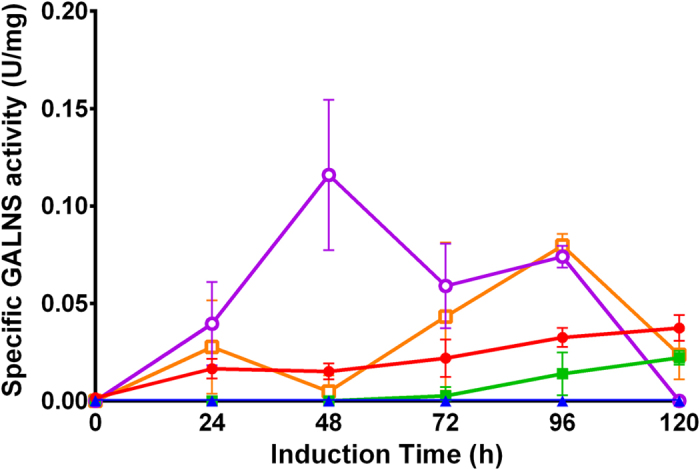
GALNS activity at 100 mL of *P. pastoris* GS115 clones transformed with pPIC9-GALNS or pPIC9-nspGALNS. All *P. pastoris* GS115 transformed clones were screened at 10 mL and those with the highest enzyme activity were selected for evaluation at 100 mL. pPIC9-GALNS/3 (filled square), pPIC9-GALNS/5 (filled circle), pPIC9-nspGALNS/1 (empty circle), pPIC9-nspGALNS/2 (empty square). *P. pastoris* GS115 transformed with empty pPIC9 vector (filled triangle) was used as control. Each clone was analyzed by triplicate.

pPIC9-GALNS/3 and 5, and pPIC9-nspGALNS/1 and 2 were selected for evaluation at 100 mL scale (Fig. 1). At this scale, pPIC9-GALNS/3 and 5 clones showed maximum enzyme activities of 0.022 and 0.037 U mg^−1^, respectively; while for pPIC9-nspGALNS the enzyme activities were 0.11 and 0.079 U mg^−1^ for clones 1 and 2, respectively. In this sense, pPIC9-GALNS/5 and pPIC9-nspGALNS/1 clones were selected for evaluation at bioreactor scale. GALNS enzyme activity was not detected at the intracellular fraction neither for pPIC9-GALNS/5 nor for pPIC9-nspGALNS/1 clones, which suggests that the α-factor secretion signal efficiently secreted the recombinant enzyme.

**Figure 2:**
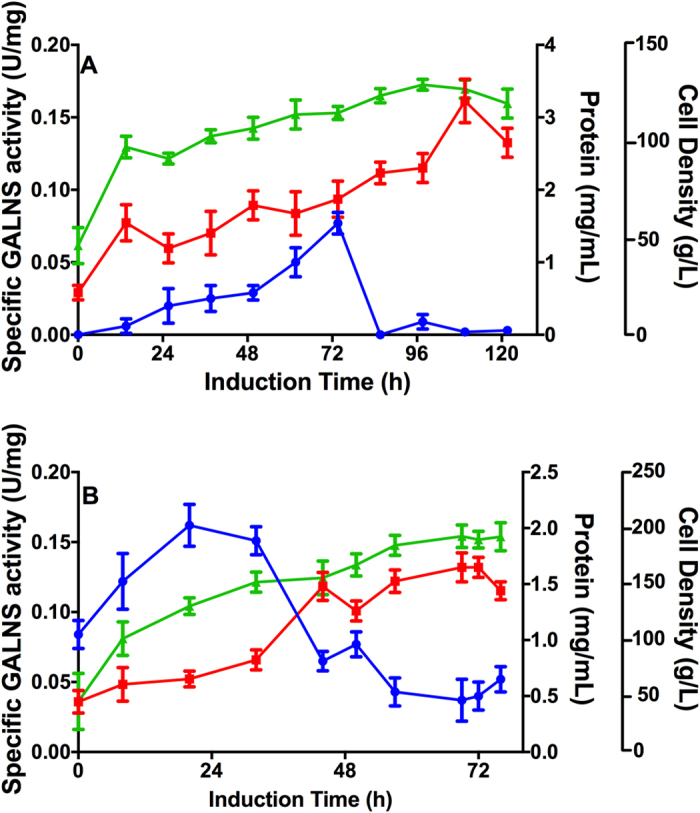
Production of prGALNS at 1.7 L scale. Production of recombinant GALNS by clones pPIC9-GALNS/5 (**A**) and pPIC9-nspGALNS/1 (**B**) was evaluated at bioreactor scale (1.7 L). Specific GALNS activity (U/mg, circle), total protein concentration (mg/ml, square), and cell density (g/L, triangle).

At bioreactor scale, pPIC9-GALNS/5 showed a final biomass of 127.6 g L^−1^ and a maximum GALNS activity of 0.077 U mg^−1^ at 73 h of induction (Fig. 2A); while pPIC9-nspGALNS/1 showed a final biomass of 189.7 g L^−1^ and a maximum GALNS activity of 0.16 U mg^−1^ at 20 h of induction, after which a marked reduction was observed (Fig. 2B). Yield of extracellular protein production was 26.4 and 8.3 mg of protein per g of biomass for pPIC9-GALNS/5 and pPIC9-nspGALNS/1, respectively, which suggest that although pPIC9-GALNS/5 produced a higher amount of total protein than pPIC9-nspGALNS/1, a higher proportion of active GALNS could be expected after removal of the native signal peptide (i.e. pPIC9-nspGALNS/1). Gene copy number in pPIC9-GALNS and pPIC9-nspGALNS strains was calculated by qPCR, and normalized against a single-copy gene (see Materials and Methods). Both pPIC9-GALNS and pPIC9-nspGALNS strains showed 1 *GALNS* gene copy number. In this sense, since both strains have similar *GALNS* copy number; these results suggest that removal of native signal peptide allowed to obtain a higher amount of secreted active recombinant GALNS.

### Production of prGALNS with SUMF1 co-expression

*SUMF1* over-expression in mammalian cells or animal models has shown a significant increase in the activity of sulfatases^4^,^6^,^32^,^33^, and in the yield of production of recombinant lysosomal enzymes for ERT^7^. In this sense, human SUMF1 cDNA was subcloned into pPIC9 vector and used to transform pPIC9-GALNS/5 and pPIC9-nspGALNS/1. Since pPIC9-GALNS and pPIC9-nspGALNS were inserted into *AOX1* locus, pPIC9-SUMF1 was linearized with *Sal*I, which cut within the *His4* gene, in order to favour vector insertion in this locus.

**Figure 3:**
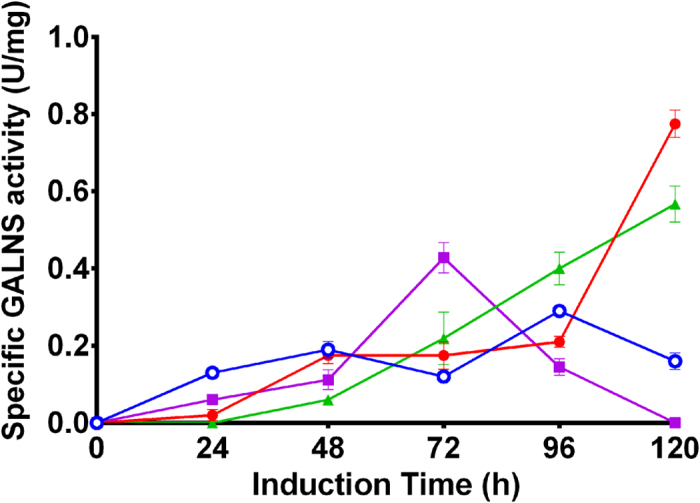
GALNS activity at 100 mL of *P. pastoris* GS115 clones co-transformed with SUMF1 and GALNS/nspGALNS. Clones of *P. pastoris* GS115 expressing GALNS or nspGALNS that showed the highest enzyme activity were transformed with pPIC9-SUMF1. Co-transformed clones were screened at 10 mL and those with the highest enzyme activity were selected for evaluation at 100 mL. Presence of SUMF1 was confirmed by PCR. pPIC9-GALNS/5-SUMF1 (empty circle), pPIC9-nspGALNS/1-SUMF1/1 (filled circle), pPIC9-nspGALNS/1-SUMF1/4 (triangle), and pPIC9-nspGALNS/1-SUMF1/5 (square). Each clone was analysed by triplicate.

**Figure 4:**
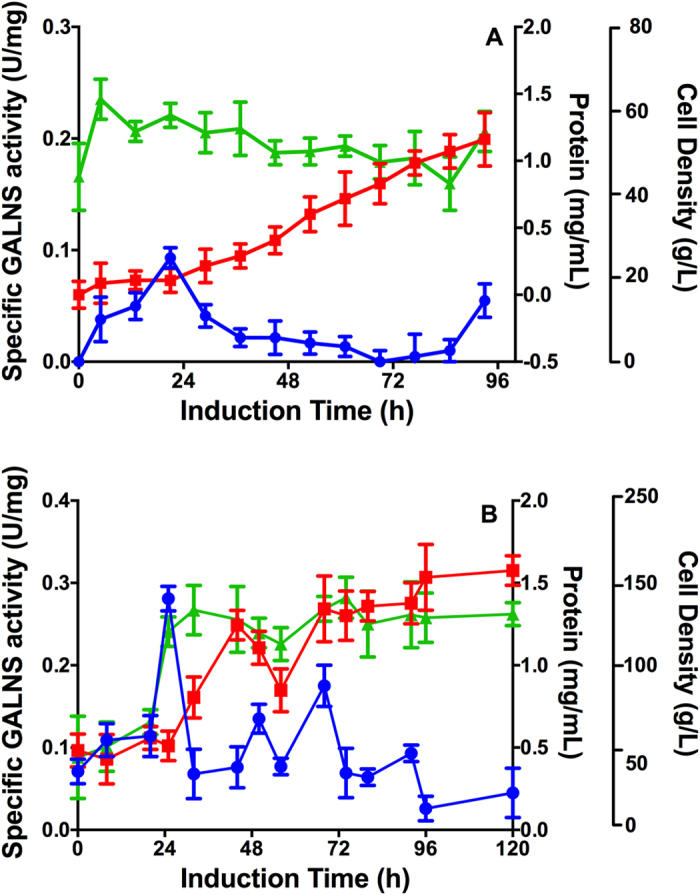
Production of prGALNS under co-expression of SUMF1 at 1.7 L scale. Production of recombinant GALNS by clones pPIC9-GALNS/5-SUMF1 (**A**) and pPIC9-nspGALNS/1-SUMF1/1 (**B**) was evaluated at bioreactor scale (1.7 L). Specific GALNS activity (U/mg, circle), total protein concentration (mg/ml, square), and cell density (g/L, triangle).

One out of five clones obtained after transformation of pPIC9-GALNS/5 with pPIC9-SUMF1 showed the presence of SUMF1 cDNA by PCR (hereafter pPIC9-GALNS/5-SUMF1), which also showed a Mut^+^ phenotype. Evaluation at 100 mL scale showed a maximum extracellular enzyme activity of 0.29 U mg^−1^ at 96 h of induction (Fig. 3), which was higher than that of pPIC9-GALNS/5 without SUMF1 co-expression. At bioreactor scale, pPIC9-GALNS/5-SUMF1 showed a final biomass of 61.1 g L^−1^ and a maximum enzyme activity of 0.09 U mg^−1^ (Fig. 4A), which was 1.3-fold higher than that of pPIC9-GALNS/5 (0.069 U mg^−1^) at this scale. The yield of extracellular protein production was 68.4 mg of protein per g of biomass.

Five out of eight clones obtained after transformation of pPIC9-nspGALNS/1 with pPIC9-SUMF1 showed the presence of SUMF1 cDNA by PCR (hereafter pPIC9-nspGALNS/1-SUMF1), and an expected Mut^+^ phenotype. At 10 mL, the highest GALNS activities were 0.24 (48 h), 0.11 (96 h), 0.13 (96 h) and 0.15 (24 h) U mg^−1^ for clones 1, 3, 4 and 5, respectively, while not enzyme activity was observed for clone pPIC9-nspGALNS/1-SUMF1/2. Clones pPIC9-nspGALNS/1-SUMF1/1, /4 and /5, were evaluated at 100 mL, with maximum GALNS activities of 0.77 (120 h), 0.57 (120 h), and 0.42 (72 h) U mg^−1^, respectively (Fig. 3). Although at 10 mL pPIC9-nspGALNS/1-SUMF1 clones did not show higher enzyme activity than that of pPIC9-nspGALNS/1 (0.39 U mg^−1^), at 100 mL scale there was an up-to 1.8-fold increment in GALNS enzyme activity. Since pPIC9-nspGALNS/1-SUMF1/1 showed the highest enzyme activity levels at 100 mL scale, this clone was selected for evaluation at bioreactor scale. At this scale, pPIC9-nspGALNS/1-SUMF1/1 showed a final biomass of 157 g L^−1^, and 1.5 mg mL^−1^ of total protein concentration. The highest enzyme activity was observed after 24 h of induction with 0.28 U mg^−1^ (Fig. 4B), which was 1.8-fold higher than the levels observed without SUMF1 co-expression (0.16 U mg^−1^). The yield of extracellular protein production was 10.4 mg of protein per g of biomass.

Taken together, these results show that co-expression of SUMF1 allowed an increase in the amount of active recombinant GALNS enzyme. Furthermore, these results showed that although *P. pastoris* GS115 can activate human GALNS, the co-expression of SUMF1 allows an up to 1.8-fold increment in enzyme activity, showing for the first time the benefit of this co-expression in a yeast expression system. These results showed that deletion of native signal peptide and co-expression of SUMF1 allowed a total improvement of 4.5-fold in specific GALNS activity.

### Purification and characterization of prGALNS

**Figure 5:**
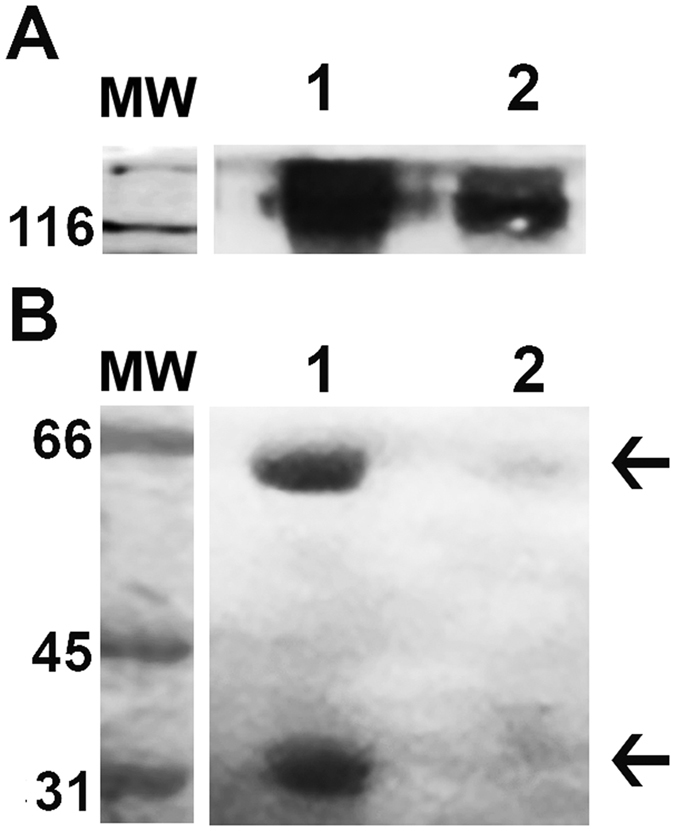
Immunodetection of recombinant GALNS. prGALNS was detected by western-blot using a polyclonal rabbit anti-GALNS IgG antibody. GALNS was detected in a purified sample of prGALNS (1) and in human leucocytes (2) under non-reduced (**A**) and reduced (**B**) conditions. Molecular weight marker (MW) was running under the same electrophoresis conditions of prGALNS and human leucocytes.

prGALNS was purified from conditioned medium through a two-column process. After purification, prGALNS showed an activity of 16.7 U mg^−1^, with a yield of 10% and a 1294.1-fold of purification (Table 1). The yield of production of prGALNS was of 1.0 mg per liter of culture. Under non-reducing conditions a band of ~120 kDa was detected by western-blot both for prGALNS and human GALNS from human leucocytes, while under reducing conditions bands of ~57 and ~39 kDa were detected both for prGALNS and GALNS from human leucocytes (Fig. 5).

**Figure 6:**
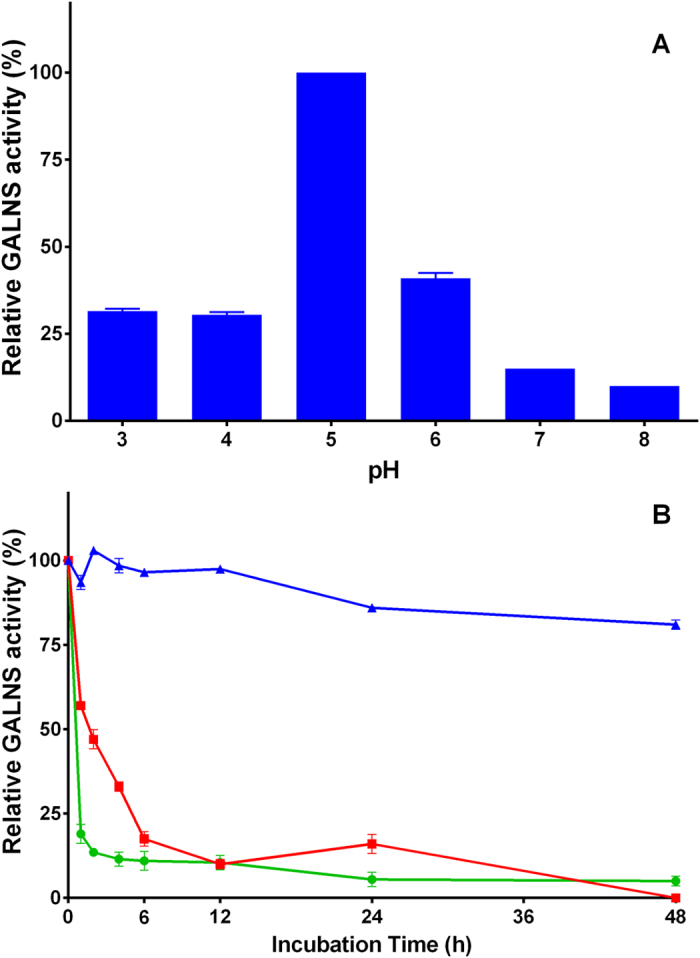
pH and temperature stability. (**A**) Recombinant GALNS was incubated at pH 3.0, 4.0, 5.0, 6.0, 7.0, and 8.0 during 1 h, after which GALNS activity was measured using the fluorogenic substrate. The activity is reported as relative activity against the enzyme activity values at pH 5.0, which was the pH of maximum enzyme activity. (**B**) Recombinant GALNS was incubated at 4 (triangle), 37 (square), and 45 (circle) °C, and the enzyme activity was monitored after 0, 1, 2, 4, 6, 12, 24, and 48 h of incubation. The activity is reported as relative activity against the activity at time 0 h. Each assay was done in triplicate.

The activity of the purified prGALNS was analysed after enzyme incubation at different pH and temperatures (Fig. 6). prGALNS showed the highest stability at pH 5.0, with a reduction in enzyme activity of up to 60% and 90% at pH values below and above 5.0, respectively (Fig. 6A). Temperature stability assay showed that prGALNS was stable at 4 °C during the evaluated time, while at 37 and 45 °C a marked reduction on the enzyme activity observed during the first 12 h of incubation, with almost complete loss of the activity after 48 h of incubation (Fig. 6B).

### Cell up-take of prGALNS

**Figure 7:**
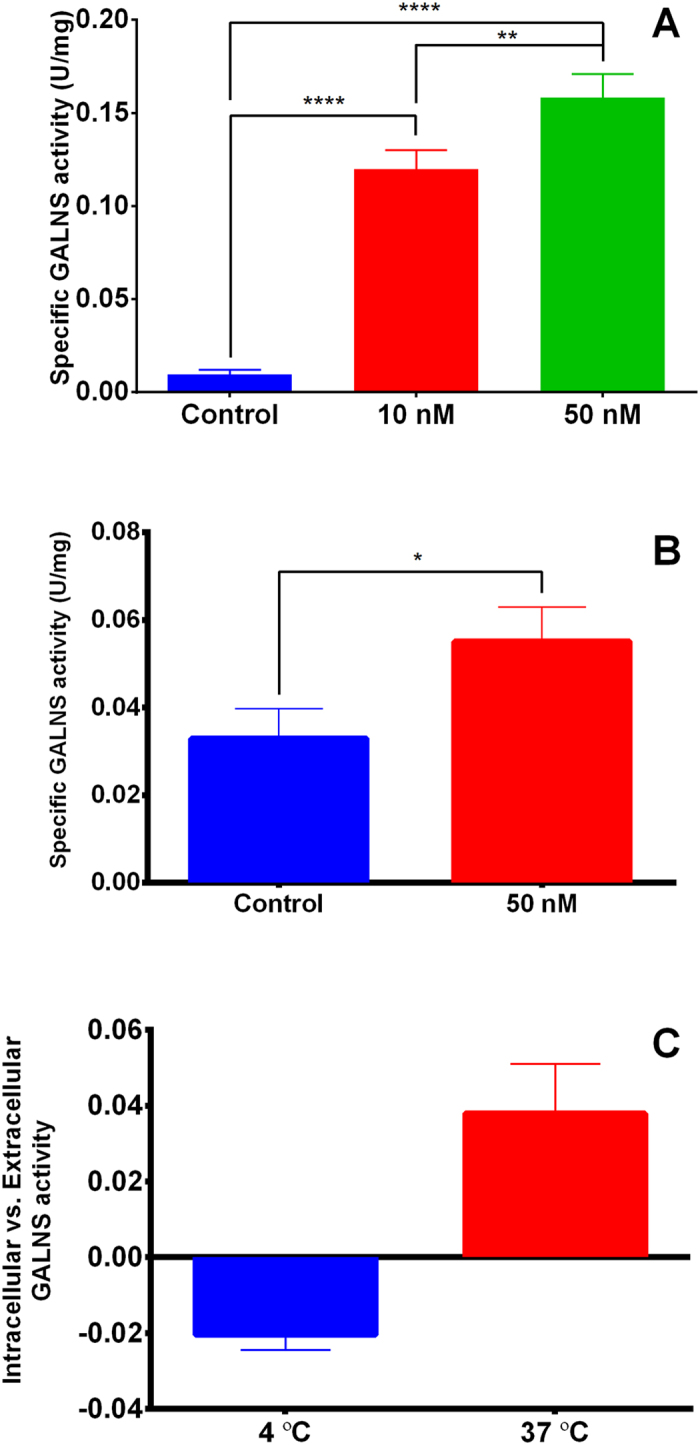
Cellular uptake. The cellular uptake of prGALNS was assayed in cultured HEK 293 (**A**) and normal human skin fibroblasts (**B**). The purified prGALNS was added to a final concentration of 0, 10 and 50 nM. (**C**) To evaluate if protein uptake was mediated through an endocytic pathway, cell uptake assay was carried out at 4 °C or 37 °C. Results at 4 °C or 37 °C are reported as the relation of intracellular *vs.* extracellular activity. In all cases the enzyme activity was assayed in the cell lysate after 5 h of incubation. Assays were done by triplicate.

Previously it was showed that recombinant GALNS produced in CHO cells were taken-up human skin fibroblasts and mouse and human chondrocytes^8^,^34^, while cellular uptake was not observed for the recombinant GALNS produced in *E. coli*^30^. In order to evaluate the potential of prGALNS towards the development of an ERT for Morquio A, the cell up-take of prGALNS was evaluated on HEK293 cells and normal human skin fibroblasts. prGALNS was added to a final concentration of 10 and 50 nM, according to previous reports for recombinant GALNS produced in CHO cells and *E. coli*^8^,^30^. As show in Fig. 7A, it was observed a significant (*P* < 0.05) and dose-dependent increase in the intracellular GALNS activity in HEK293 cells. Similarly, addition of 50 nM prGALNS in human skin fibroblasts produced a significant increase in the intracellular GALNS activity (Fig. 7B), though this increment (1.7-fold) was lower than that observed in HEK293 cells with the same prGALNS concentration (15.6-fold).

It is known that cellular internalization processes occur at physiological temperatures, while low temperatures can affect or even inhibit the endocytic pathway^35^. In this sense, to evaluate if the cellular uptake of prGALNS was mediated by an endocytic pathway, the cellular uptake assay was carried out at 4 °C and compared against the results observed at 37 °C. As observed in Fig. 7C, at 4 °C most of the enzyme was detected extracellularly, while at 37 °C the enzyme was taken-up, suggesting that the cellular capture of this recombinant enzyme could be mediated by an endocytic pathway. Taken together, these results strongly suggest that N-glycosylations synthetized by *P. pastoris* allow the cell up-take of recombinant GALNS, and that this process could be mediated by an endocytic pathway as has been observed for other lysosomal enzymes produced in yeast^36^.

## Discussion

Currently, the main treatment alternative for Morquio A patients is the use of ERT through the infusion of Elosulfase alfa. This recombinant GALNS, produced in CHO cells, was approved on 2014 and since then several studies have shown that this enzyme is a therapeutic option for the treatment of Morquio A disease^10^,^37^. However, several limitations have been observed for this ERT, including a limited effect on skeletal, corneal, and heart valvular issues;^10^,^11^,^37^ a short half-life (40 min) of the enzyme and rapid clearance from the circulation^38^; immunological issues^14^; and a high cost. Microorganism has emerged as an alternative to the conventional production of lysosomal enzymes in CHO cells^15^, since production in microorganisms is considerably less expensive than in mammalian cells and, consequently, may significantly reduce the cost of ERT. In addition, microorganism can be engineered to producing recombinant proteins with improved stability, and pharmacokinetic and pharmacodynamics profiles^39^. In this study, we report the production of an active recombinant GALNS in *P. pastoris* (prGALNS), showing that this enzyme has a similar stability profile to previous recombinant GALNS and that it can be taken up by cultured cells without any further modification.

An important observation in all cultures at bioreactor scale was the detection of enzyme activity at 0 h of induction (Figs 2 and 4). Since expression of recombinant GALNS was under the regulation of *AOX1* promoter, recombinant protein was not expected before the addition of methanol. *AOX1* promoter is repressed during growth phase in glucose, glycerol, or ethanol; de-repressed after depletion of carbon source, and fully induced upon the addition of methanol^40^. In this sense, we consider that enzyme activity detected at 0 h of induction could be the result of a depletion of the carbon source (i.e glycerol) before the culture reached the expected induction biomass^41^. In fact, at bioreactor scale, we observed that glycerol was depleted about 2 h before the culture reached 60 g L^−1^ (Supp. Fig. 1), which is the suggested biomass for high cell density fermentation^41^.

The results showed that through the deletion of native signal peptide and co-expression of human *SUMF1* gene it was possible to obtain an increment in GALNS activity. First, a 2.1-fold increment in the extracellular enzyme activity was observed after removal of native GALNS signal peptide. This result agrees with previous reports showing that the presence of both α-factor secretion signal from *Saccharomyces cerevisiae* and native signal peptide decrease the production of the recombinant enzyme. For instance, Barragan *et al*.^42^, through a systematic review of the literature, suggested that the presence of the native signal peptide downstream of the α-factor secretion signal, might affect the maturation and secretion of the recombinant enzymes produced in *P. pastoris*, as well as the recognition of the secretion signal by the signal peptidase I and Kex2. Similarly, recombinant human interferon-γ was not detected in *P. pastoris* X-33 when interferon cDNA with the native signal peptide was inserted into the expression vector downstream of the α-factor^43^. Production of thermostable alkaline protease from *Bacillus stearothermophilus* F1 in two different *P. pastoris* strains (GS115 and SMD1168H) was about 1.5-fold higher when the open reading frame was inserted into the expression vector without the native signal peptide^44^. This effect on maturation and secretion of the heterologous protein due to the presence of both native signal peptide and α-factor secretion signal, could also explain the longer induction time require for pPIC9-GALNS strains to reach the highest enzyme activity levels in comparison with that observed for pPIC9-nspGALNS strains.

On a second stage, we evaluated the co-expression of *SUMF1* that encodes for the formylglycine-generating enzyme (FGE), since this has been identified as an essential factor for the maturation of human sulfatases^4^,^6^,^32^,^33^,^45^,^46^. Production of prGALNS by co-expressing *SUMF1* led to an increase of up to 1.8-fold in the specific GALNS activity, which is compatible with previous results of *SUMF1* co-expression. For instance, *GALNS* and *SUMF1* co-expression for gene therapy showed enzyme activity levels between 2.0- and 4.5-fold higher than those levels observed without *SUMF1* co-expression, depending on the transduced cell^6^. On the other hand, production of recombinant GALNS in CHO cells with the co-expression of *SUMF1* led to a 1.2-fold increment in the enzyme activity^7^; while the production of a recombinant iduronate-2-sulfatase in COS cells with the co-expression of *SUMF1* led to 5-fold higher enzyme activity levels than those observed without *SUMF1* co-expression^47^. Furthermore, at bioreactor scale we observed a fluctuation in enzyme activity, which could be associated to protease activity or GALNS activation process. In particular, GALNS activation could have an important impact in this fluctuation, since Cys-to-FGly conversion is an essential and limiting factor in maturation of sulfatases^46^.

Recombinant GALNS enzyme for ERT has been produced in CHO cells, exhibiting after purification a specific activity of 170,000^34^ to 120,000 U mg^−1 ^8. Both enzymes showed the highest activity at pH 5.0; contain 57, 39, and 19 kDa polypeptides; and are taken-up by cultured fibroblast and chondrocytes^7^,^8^. The production of recombinant GALNS in microorganisms was first reported in *E. coli* BL21(DE3) (erGALNS)^29^,^30^,^31^. At 100 mL scale, the highest enzyme activity was between 0.054 and 0.071 U mg^−1 ^29, while at 3 L scale GALNS activity was up-to 6.18 U mg^−1 ^30. The purified GALNS showed a specific activity of 0.29 nmol h^−1^ mg^−1^, an optimal pH of 5.5, and it was stable for 8 days at 4 °C and 6 h in human serum^30^. Western-blot analysis showed that recombinant GALNS produced in *E. coli* is a polypeptide of ~50 kDa^29^.

In the present study, purified prGALNS showed a final enzyme activity of 16.7 U mg^−1^, which was higher than that of *E. coli* but lower than the enzyme produced in CHO cells. However, GALNS activity values between recombinant enzyme produced in microorganisms (bacterial and yeast) and CHO cells are not equivalent since they have been obtained using substantially different methods (e.g. substrate concentration or protein immune capture)^48^. Nevertheless, similarly to GALNS produced in CHO cells and *E. coli*, prGALNS showed the highest stability at pH 5.0, while at higher or lower pH values the activity decreased markedly. However, these results differ from those of GALNS purified from human placenta and liver, which showed an optimal pH between 3.5 and 4.0 with a linear decrease in enzyme activity at higher pH values 49,50. prGALNS showed a high stability at 4 °C during the evaluated time, similar to the reported stability profile for GALNS produced in *E. coli*^30^, GALNS purified from human placenta^51^, and recombinant GALNS produced in CHO cells^34^. However, at 37 °C prGALNS showed lower stability than that reported for erGALNS^30^, since no activity was detected for prGALNS after 48 h of incubation, while a 45% residual activity was still observed for erGALNS after 8 days of incubation. This difference in stability between recombinant GALNS produced in *E. coli* and *P. pastoris*, could be due to the N-glycosylations presence on prGALNS. In this sense, it has been reported that the presence and structure of N-glycans can affect the function, folding, and stability of a protein^52^ and that yeast N-glycosylations can affect the thermolability of recombinant enzymes produced in this host^53^. Unfortunately, there are not reports about the stability at 37 °C for GALNS from tissues or produced recombinantly in mammalian cells. Finally, at 45 °C prGALNS showed a 80% reduction on the activity after 1 h incubation, which agrees with the results reported for GALNS purified from human placenta that showed a 50% reduction after 30 min incubation at 50 °C^51^. Taken together, these results suggest that prGALNS has a similar pH and temperature stability profile to that reported for native or recombinant GALNS isolated from mammalian tissues or cells.

Finally, we evaluated the cell uptake of the recombinant enzyme by HEK293 cells and normal fibroblasts. The results showed that prGALNS was taken-up by cultured cells in a dose-dependent manner, without any additional modification on the structure of the N-glycosylations. In this sense, these results contrast with those of recombinant Hex-A^54^,^55^, α-glucosidase^56^, and α-galactosidase A^57^ produced in the yeasts *O. minuta, Y. lipolytica,* and *S. cerevisiae*, respectively, which required the treatment with a bacterial glycosidase to remove the terminal α-1,2-mannose, uncap the M6P residues, and allow the cell uptake. However, the present results are in agreement to those reported for human α-glucosidase^18^, lysosomal acid lipase^24^,^28^, and β-hexosaminidases^58^ produced in *P. pastoris*, which showed a dose-dependent cell uptake without any additional processing of the enzymes. N-glycosylations analysis for the lysosomal acid lipase produced in *P. pastoris* (phLAL) showed high mannose N-glycans (Man_8–12_)^24^, similar to those reports for other recombinant enzymes produced in yeasts^26^. Furthermore, phLAL showed mono- and dephosphorylated oligomannosyl structures, in a similar ration to that observed for lysosomal recombinant enzymes produced in CHO cells^24^. This phLAL was taken up through mannose receptors and targeted to the lysosome^28^. *In-vivo* evaluation of phLAL showed that it was taken-up by liver and spleen cells, and that it has similarly biodistribution profile to that observed for a recombinant mannose-terminated lysosomal enzyme produced in mammalian cells^28^. In this sense, we expect that prGALNS has similar N-glycosylations structures to those observed for phLAL, suggesting that the enzyme could be taken up through mannose receptors and targeted to the lysosomal. Although further *in-vitro* and *in-vivo* experiments are necessary, these results strongly suggest that prGALNS could be used for the development of an ERT for Morquio A disease. In addition, these results also present valuable data to continue exploring *P. pastoris* as a platform for the production of recombinant lysosomal enzymes for ERT.

## Conclusions

In this study, we report for the first time the production and characterization of the human recombinant GALNS produced in the yeast *P. pastoris* GS115. We observed that removal of native signal peptide and co-expression with the human formylglycine-generating enzyme allowed a total improvement of 4.5-fold in specific GALNS activity. In this sense, this study represents the first report of the benefit sulfatse-SUMF1 co-expression in a yeast expression system. The prGALNS showed a similar post-translational process to that of GALNS from human leucocytes, and the enzyme showed the highest activity at pH 5.0 and a high stability at 4 °C. It was noteworthy that the prGALNS was taken-up by cultured cells in a dose-dependent manner through a process potentially mediated by an endocytic pathway. The results show the potential of *P. pastoris* in the production of a human recombinant GALNS for the development of an ERT for Morquio A, which could be extended to other human lysosomal enzymes.

## Methods

### Expression vectors and yeast transformation

Native human GALNS cDNA (GenBank accession number NM_000512.4), with or without (nspGALNS) signal peptide coding sequence, was inserted in pPIC9 plasmid (Life Technologies, Grand Island, NY, USA) to produce the expression vectors pPIC9-GALNS and pPIC9-nspGALNS, respectively. Vectors were linearized and used to independently transform competent cells of *P. pastoris* GS115. A *P. pastoris* GS115 transformed with an empty pPIC9 vector was used as a negative control. The *P. pastoris* pPIC9-GALNS and pPIC9-nspIDS clones with the highest GALNS activity were transformed with the expression vector pPIC9-SUMF1, which encodes for the human formylglycine-generating enzyme. Gene insertion was confirmed by PCR using the primers TOMF23 5′-acagggccattgatggcctcaacctcct-3′ and TOMF34R 5′-gcttcgtgtggtcttccagattgtgagttg-3′, which amplify a 235 bp fragment of human GALNS cDNA, and SUMF1-F-Topo 5′-caccatggctgcgcccgcacta3′ and SUMF1-R-Topo 5′-aaagtccatagtgggcaggcg-3′, which amplify a 1 kb fragment of human SUMF1 cDNA. Phenotype of *P. pastoris* clones was confirmed by PCR using the primers FW 5′-gactggttccaattgacaagc-3′and RW 5′-gcaaatggcattctgacatcc-3′ (Life Technologies Corporation), as previously reported^59^. All procedures were carried out under standard molecular biology methods^60^.

### Gene copy number determination using qPCR

Gene copy number determination was carried out in pPIC9-GALNS and pPIC9-nspGALNS strains as previously described^61^,^62^. Briefly, reaction mixture was prepared using LightCycler FastStar DNA Master^PLUS^ SYBR Green I kit (Roche Diagnostics GmBH, Mannheim, Germany), following manufacturer instructions. Samples and standards were measured in triplicate. *GALNS* was amplified with primers TOMF23 and TOMF34R, described above; while ORF2 from *PDI* gene (GenBank AJ302014.1) was selected for the reference amplification reaction with primers ORF2_F 5′-TTCGAAGGATCCATGACTAACTGGAAAGCGATATTGAC-3′ ORF2_R 5′-CCTAGGGAATTCTTAGTTCTCTTCTTCACCTTGAAATTTTAGG-3′. To verify the specificity of the amplicons, a melting curve was generated from 65 to 95 °C, with 0.5 °C increments/cycle^61^.

### Shake flask cultures

Screening of *P. pastoris* clones was done at 10 and 100 mL. Clones were grown in YPD medium (Yeast extract 1% p/v; Peptone 2% p/v; Dextrose 2% p/v) during 48 hours at 28 °C and 250 RPM. Cells were harvested by centrifugation and the pellet was resuspended in BMG medium (potassium phosphate 100 mM pH 6.0; yeast nitrogen base 1.34%; biotin 4 × 10–5%; glycerol 1%) and cultured for 24 hours at 28 °C and 250 RPM. Finally, cells were recovered and resuspended in BMM medium (potassium phosphate 100 mM pH 6.0; yeast nitrogen base 1.34%; biotin 4 × 10^−5^%; glycerol 1%; methanol 0.5%), and cultured for 120 hours at 28 °C and 250 RPM. Methanol was added every 24 h to maintain a final concentration of 0.5%. Aliquots were taken every 24 h and stored at −20 °C until their use. Clones with the highest activity at shake flask were selected for evaluation at bioreactor scale. Aliquots were centrifuged and GALNS activity was measured in the supernatant. The intracellular activity was measured only in samples from 100 mL scale. Briefly, aliquots were centrifuged and pellet was resuspended in lysis buffer (Tris-HCl 0.025 M pH 7.2, EDTA 1 mM, PMSF 1 mM, DTT 2 mM), and cell lysis was done by using glass beads with 15 cycles of 1 min vortex/1 min 4 °C, followed by 8 cycles (15 sec on/45 sec off) of sonication with 25% amplitude (Vibra-Cell, Sonics & Materials Inc., Newtown, CT, USA). Cell debris was removed by centrifugation and GALNS activity was measured in the supernatant.

### Bioreactor cultures

Bioreactor production of recombinant GALNS was done at 1.65 L scale in a KLF 3.7 L Bioengineering reactor. Cultures were done in modified fermentation medium FM22 (composition per liter: KH_2_PO_4_ 25.74 g, (NH_4_)_2_SO_4_ 3 g, K_2_SO_4_ 8.58 g, CaSO_4_ 2H_2_O 0.6 g, glycerol 40 g, MgSO_4_ 7H_2_O 7.02 g, Biotin 4 × 10^−5^% w/v, supplemented with Pichia trace minerals PTM4 1.0 mL)^41^. Protein production was done in two phases: i) a batch culture with glycerol to achieve 60 g L^−1^ biomass, and ii) a fed-batch induction phase with methanol, which was maintained at 0.5% by using an ALCOSENS probe (Heinrich Frings GmbH & Co. KG), with an automatic feed control. Cultures were done at 28 °C and pH 5.0, under limited oxygen conditions^63^, during 96 h. Aliquots were taken every 8 to 12 h, and stored at −20 °C until their use. The cell density (g DCW L^−1^) was determined at 600 nm by using a previous reported calibration curve for *P. pastoris* GS115^20^. Aliquots were centrifuged and GALNS activity was measured in the supernatant

### Crude protein extracts and enzyme purification

prGALNS was purified from a culture medium of a pPIC9-nspGALNS with SUMF1 co-expression culture. Culture medium (~1.7 L) was filtered sequentially through Whatman™ paper No. 1 and 42, and 0.45 and 0.22 μm polyether sulphone membranes (Pall Corp, Port Washington, NY, USA). Permeate was ultra-filtered through a 30 kDa cut-off membrane (Millipore, Billerica, MA, USA), up to reach a final volume of 20 mL, and adjusted to pH 4.5 with 25 mM sodium acetate. Finally, the retentate was dialyzed overnight at 4 °C against 25 mM sodium acetate buffer (pH 4.5) with constant stirring. prGALNS was purified by a two-step chromatography process as previously described for recombinant GALNS produced in *E. coli*^30^. Briefly, a cation exchange chromatography was performed with a Macro-Prep High S support column (Bio-rad, Hercules, CA, USA), equilibrated with 25 mM potassium acetate, pH 4.5, and eluted with a linear gradient of 0–0.5 M NaCl. Fractions with GALNS activity were pooled and applied to a Sephacryl™ S-200 High Resolution column (GE Healthcare, Uppsala, Sweden) with 25 mM potassium acetate, pH 4.5, 10 mM NaCl, in a flow rate of 0.5 mL min^−1^. Finally, fractions with the highest GALNS activity were pooled and lyophilized. Protein purification was monitored by SDS–PAGE under reducing conditions and GALNS enzyme activity.

### Enzyme activity

GALNS activity was assayed by using 4-methylumbelliferyl-β-d-galactopyranoside-6-sulfate (Toronto Chemicals Research, North York, ON, Canada) as substrate^64^. One unit (U) was defined as the amount of enzyme catalyzing 1 nmol substrate per hour. Specific GALNS activity was expressed as U mg^−1^ of protein as determined by Bradford assay. At bioreactor scale, total units of recombinant GALNS were calculated as the sum of units (nmol h^−1^) produced at each sampling time.

### Western-blot

Crude protein extracts and purified fractions were analysed by sodium dodecyl sulfate (SDS) polyacrylamide gel electrophoresis (PAGE) under reducing and non-reducing conditions. Samples were electrotransferred to a nitrocellulose membrane (Hybond C-Extra; Amersham Bioscience, Piscataway, NJ, USA). The recombinant GALNS was recognized with a polyclonal rabbit anti-GALNS IgG antibody, produced against a mixture of highly immunogenic human GALNS peptides^29^, followed by incubation with an anti-rabbit IgG coupled with peroxidase (Sigma-Aldrich). Peroxidase activity was visualized using diaminobenzidine substrate (Sigma–Aldrich). A leucocytes lysate from a human unaffected donor was used as positive control.

### prGALNS characterization

Effect of temperature and pH on GALNS was assayed as previously reported for the recombinant GALNS produced in *E. coli*^30^. To evaluate the temperature stability of prGALNS, 20 μL of purified enzyme were independently incubated with 20 μL of 50 mM sodium acetate pH 5.5 at 4, 37, and 45 °C for 1, 2, 4, 6, 12, 96, 120, and 192 h. To evaluate the effect of pH on prGALNS stability, 20 μL of the purified enzyme were incubated with 20 μL of 50 mM sodium citrate pH 3.0, or 50 mM sodium acetate pH 4.0, 5.0, 5.5, or 6.0, or 50 mM Tris–HCl pH 7.0 or 8.0, respectively. Samples were incubated at 37 °C for 1 h. In all cases, after incubation under the respective conditions, the enzyme activity was assayed as previously described.

### *In vitro* evaluation of recombinant GALNS

The cellular uptake of prGALNS was assayed in cultured HEK 293 (ATCC CRL1573) and normal human skin fibroblasts, as previously described^30^. Cells were cultivated in Dulbecco’s modified medium (DMEM, Gibco, Carlsbad, CA), supplemented with fetal bovine serum 15% (Eurobio, Les Ulis, Francia), penicillin 100 U mL^−1^ and streptomycin 100 U mL^−1^, at 37 °C in a CO_2_ incubator. Twenty-four hours before the experiment, 1 × 10^5^ cells per well were seeded in 12-well plates, and the culture medium was replaced with fresh medium 2 h before the experiment. The purified enzyme was added to a final concentration of 0, 10 and 50 nM^8^. After 5 h of incubation the culture medium was removed, and the cells were washed three times with cold PBS 1x (composition per liter: 8 g NaCl, 0.2 g KCl, 1.44 g NaH_2_PO_4_, 0.24 g KH_2_PO_4_, pH 7.2). Cells were lysed by resuspension in 1% sodium deoxycholate (Sigma-Aldrich). The enzyme activity was determined in the cell lysate and the culture medium as described above. Finally, to evaluate if protein uptake was mediated through an endocytic pathway, cell uptake assay was carried out at 4 °C or 37 °C with recombinant proteins at a final concentration of 50 nM. Enzyme activity was assayed in the culture medium and cell lysates as described above. Assays were done by triplicate. The experiments were approved by the Research and Ethics Committee of the School of Science at Pontificia Universidad Javeriana.

### Statistical analysis

Differences between groups were tested for statistical significance by using Student’s t-test or one-way ANOVA. An error level of 5% (p < 0.05) was considered significant. All analyses were performed using SPSS v21.0 (SPSS Inc., Chicago, IL, USA). All results are shown as mean ± SD.

## Additional Information

**How to cite this article**: Rodríguez-López, A. *et al*. Recombinant human N-acetylgalactosamine-6-sulfate sulfatase (GALNS) produced in the methylotrophic yeast *Pichia pastoris. Sci. Rep.* **6**, 29329; doi: 10.1038/srep29329 (2016).

## Supplementary Material

Supplementary Information

## Figures and Tables

**Table 1:** Purification of recombinant GALNS produced in *P. pastoris.*

	Protein (mg)	Units (nmol h^−1^)	Specific activity (U mg^−1^)	Yield (%)	Fold
Crude extract	2666.0	34.4	0.01	100.0	1.0
Rententate (30 kDa)	207.7	10.4	0.05	30.1	3.9
Macro-Prep High S support	0.87	6.5	7.43	18.8	576.1
Sephacryl™ S-200	0.20	3.3	16.69	9.7	1294.1
