# Supplementary material for: Recombinant human N-acetylgalactosamine-6-sulfate sulfatase (GALNS) produced in the methylotrophic yeast Pichia pastoris
Source: Sci Rep. 2016 Jul 5;6:29329. doi: 10.1038/srep29329 (PMC4932491; doi:10.1038/srep29329)
Supplement: Supplementary Information [file srep29329-s1.pdf]

SUPPLEMENTARY MATERIAL

Recombinant human N-acetylgalactosamine-6-sulfate sulfatase (GALNS) produced in the methylotrophic yeast *Pichia pastoris*

Alexander Rodríguez-López[1,2], Carlos J. Alméciga-Díaz[1,*], Jhonnathan Sánchez[1], Jefferson Moreno[1], Laura Beltran[1], Dennis Díaz[1], Andrea Pardo[1], Aura María Ramírez[1], Angela J. Espejo-Mojica[1], Luis A. Barrera[1].

[1] Institute for the Study of Inborn Errors of Metabolism, School of Sciences, Pontificia Universidad Javeriana, Bogotá, Colombia.

[2] Chemical Department, School of Science, Pontificia Universidad Javeriana, Bogotá, Colombia.

*** Corresponding author:**

Carlos Javier Alméciga-Díaz. Protein Expression and Purification Laboratory, Institute for the Study of Inborn Errors of Metabolism School of Science, Pontificia Universidad Javeriana, Cra 7 No 43 E 82, Building 54, Room 303A, Bogotá, Colombia. Tel.: +57 1 3208320 Ext. 4140. cjalmeciga@javeriana.edu.co

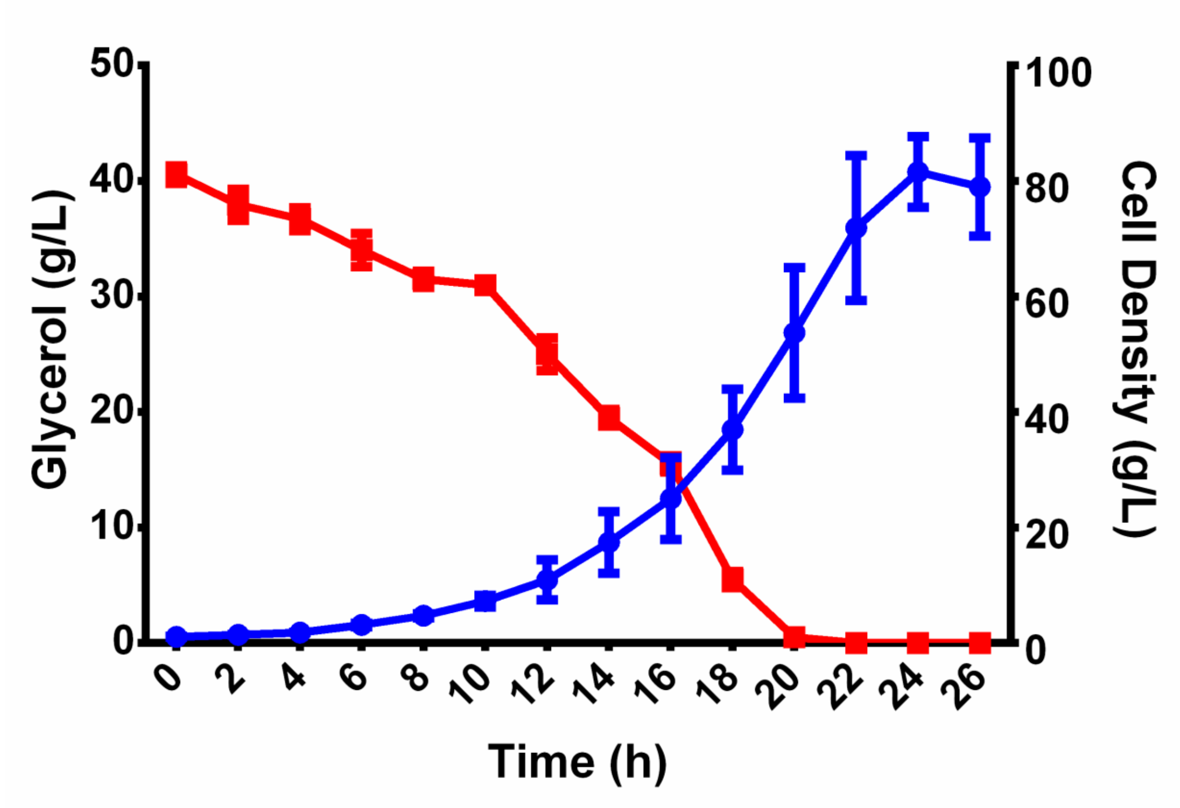

Supplementary Figure 1. Time-course evaluation of cell density (g L^{-1}) and glycerol concentration (g L^{-1}) during growth (batch) phase of *P. pastoris* GS115 pPIC9-GALNS strain. The cell density (g DCW L^{-1}) was determined at 600 nm by using a previous reported calibration curve for *P. pastoris* GS115[1]. Glycerol concentration was measured by using the glycerol phosphate oxidase/peroxidase kit (Biosystems) following manufacturer´s instructions. Similar results were obtained for the *P. pastoris* GS115 pPIC9-nspGALNS strain.

Reference.

1 Córdoba-Ruiz, H. A. *et al.* Laboratory scale production of the human recombinant iduronate 2-sulfate sulfatase-Like from Pichia pastoris. *Afr. J. Biotechnol.* **8**, 1786-1792 (2009).
